# Torch-eCpG: a fast and scalable eQTM mapper for thousands of molecular phenotypes with graphical processing units

**DOI:** 10.1186/s12859-024-05670-4

**Published:** 2024-02-14

**Authors:** Kord M. Kober, Liam Berger, Ritu Roy, Adam Olshen

**Affiliations:** 1 School of Nursing, University of California San Francisco, San Francisco, CA USA; 2 Helen Diller Family Comprehensive Cancer Center, University of California San Francisco, San Francisco, CA USA; 3 Bakar Computational Health Sciences Institute, University of California San Francisco, San Francisco, CA USA

**Keywords:** DNA methylation, Gene expression, Transcriptional regulation, Expression quantitative trait methylation, eQTM, eCpG, GPU, Tensor

## Abstract

**Background:**

Gene expression may be regulated by the DNA methylation of regulatory elements in *cis*, *distal*, and *trans* regions. One method to evaluate the relationship between DNA methylation and gene expression is the mapping of expression quantitative trait methylation (eQTM) loci (also called expression associated CpG loci, eCpG). However, no open-source tools are available to provide eQTM mapping. In addition, eQTM mapping can involve a large number of comparisons which may prevent the analyses due to limitations of computational resources. Here, we describe Torch-eCpG, an open-source tool to perform eQTM mapping that includes an optimized implementation that can use the graphical processing unit (GPU) to reduce runtime.

**Results:**

We demonstrate the analyses using the tool are reproducible, up to 18 × faster using the GPU, and scale linearly with increasing methylation loci.

**Conclusions:**

Torch-eCpG is a fast, reliable, and scalable tool to perform eQTM mapping. Source code for Torch-eCpG is available at https://github.com/kordk/torch-ecpg.

## Background

Gene expression is regulated, in part, by epigenetic mechanisms. A major unanswered question in genomics research is the functional contribution of epigenetic variation on gene expression [1, 2]. One method to evaluate for the potential functional effect of a methylation variation is to test for an association between levels of methylation and gene expression from the same samples. These expression-associated quantitative trait methylation (eQTM) loci may contribute to the regulation of gene expression (also called expression associated CpG loci, eCpG). These associations may be local (e.g., methylation located in the promoter region of a gene) or remote (e.g. methylation loci in a distant enhancer regions of a gene or on a different chromosome). There is growing interest in the integration of these data modalities and evaluating for eQTMs. For example, in terms of clinical research, recent studies have identified eQTMs from a variety of tissue types and outcomes [3–7].

Recent advances in high throughput molecular methods allow for the collection of complementary methylation and gene expression data from the same sample in large numbers. Although an increasing number of recent studies have provided eQTM datasets [3, 8], there are no open-source tools currently available to investigators to implement these analyses on their own.

Given the increase in the availability of complementary datasets and the biological utility of identifying eQTMs, analytic tools must be made available, free to use, and able to scale to handle thousands to millions of samples (e.g., patients or single cells). Current methylation array datasets provide hundreds of thousands of loci (e.g., Infinium MethylationEPIC, Illumina, San Diego, CA) and RNA-sequencing and microarray methods provide expression levels for tens of thousands of genes. An exhaustive evaluation of these datasets would result in tens of billions of tests for hundreds or thousands of samples, quickly outreaching the computing capacity of a desktop computer and requiring larger workstations, clusters, or cloud computing [9]. Future datasets will likely include more loci for evaluation and larger sample sizes. This need for computational resources will also require improvements in efficiency. Graphical processing units (GPUs) have provided major improvements in computation efficiency (i.e., runtime) for many bioinformatic software tools [10]. Readily available open source libraries implement numerous general-purpose methods and mathematical primitives that allow for major improvements in computational efficiency at relatively lower costs as compared to CPUs [11].

Given the analytic utility of evaluating for eQTMs to identify relationships between gene expression and epigenetic changes, a lack of an available open-source tool to implement an eQTM analysis, and the performance benefits of utilizing a GPU, the objectives of this project were to develop an open source, general-use tool for eQTM mapping and evaluate for performance increases of a GPU implementation. Here, we present the Torch-eCpG tool (tecpg).

## Implementation

### Association analyses

Two methods are available to test for the associations between CpG methylation and gene expression (eCpGs). First, a Pearson correlation can be computed between the methylation level and gene expression level. Second, a multivariate linear regression (MLR) method can model the relationship between gene expression and methylation level while including while adjusting for covariates (e.g., age, batch, cell type composition, population structure). Although more complex mapping approaches are available [3], this approach is commonly used and the methods are easily accessible [4, 8, 12]. Future versions of the tool may include additional mapping approaches. We tested for an association between methylation at CpG *j* and the expression level of transcript *k*, by fitting the model

1 $$y_{k} = M_{j} a_{jk} + Xb_{jk} ,\;\; \, j = 1,..,n,\;\;k = 1,..,m$$

where *y*_*k*_ is a vector of log expression levels at gene *k* with length *m*, *M*_*j*_ is a size *n* vector of methylation values (i.e., Beta scores) at CpG *j*, *m* is the number of covariates, and *X* is a *n* × *m* matrix of covariates.

Given the PyTorch toolkit does not currently include a function to estimate the cumulative distribution function (CDF) for the Student’s t-distribution, and thus it is not easily possible to compute a p-value based on the t-distribution, we used a Gaussian distribution CDF to estimate p-values. The Gaussian distribution converges to the Student’s t-distribution as the degrees of freedom (e.g., the number of samples) increases. For smaller sample sizes (e.g., < 20 samples) the difference between the t and the Gaussian distributions may have a noticeable impact. The MLR feature of tecpg is optimized to increase performance for large input datasets. Optimizations include the minimization of repeated calculations, parallelizing tasks, memory use management, data chunking, and selective use of tensors on the GPU.

Gene expression, methylation, and phenotypic (i.e., covariate) data are provided as comma-separated value (CSV) files. Gene expression and methylation data are provided with samples in columns and loci in rows. Sample metadata (i.e., phenotypic) are provided with the covariate in the columns and samples in rows. Gene and methylation loci genomic region annotations are provided as browser extensible data (BED) files [13]. Examples of annotation files are provided for the HumanMethylation450 (n = 349,220 CpG loci) and HumanHT-12 (n = 39,353 expression probes) arrays. For evaluation, the tool can either create simulated random data (i.e., `tecpg data dummy`) or download and format data from the Grady Trauma Project (GTP) [Gene Expression Omnibus (GEO) accession numbers GSE72680, GSE58137] (i.e., `tecpg data gtp`).

Four eCpG mappings modalities are implemented:

- *Cis*-eCPG: associations between all methylation loci-gene expression pairs within a specified window (default + 1 Mb) around the transcript start site for genes.
- *Distal*-eCpG: associations between all methylation loci-gene expression pairs outside of a specified window (default 50 Kb) from the transcript start site for genes, but on the same chromosome.
- *Trans*-eCpG: associations between all methylation loci-gene expression pairs. The computation is performed for each chromosome using methylation loci on all other chromosomes. To reduce output size, only associations below a given *p* value threshold (default 1 × 10^–5^) are stored.
- *All*-*by-all*: associations between all methylation loci-gene expression pairs across all regions. To reduce output size, only associations below a given *p* value threshold (default 1 × 10^–5^) are stored.

Numerous user-friendly features are provided. The tool will attempt to automatically detect a CUDA supported GPU. If a supported GPU is not available, or upon user request, the analyses will be performed using a CPU. The number of CPU threads is configurable and threaded CPU processing is available. In the case where data sizes exceed the CPU or GPU memory, the tool can be set to batch the analyses into chunks of gene expression and/or methylation data. Torch-eCpG can chunk the data for analysis as requested by the user through the settings. For users needing guidance to select settings, an option is available to estimate the number of gene expression loci per chunk. Chunking of the data was used for the evaluations described below. Finally, to limit the size of the output and associated time writing the file out, the user can set a p-value threshold to filter the reported analyses and can select the columns of the MLR analyses to report.

### Evaluation

To evaluate for the replicability of the regression analyses implemented in tecpg, we compared our regression analyses with similar analyses using the cor() and lm() functions in the stat package in R. To benchmark tecpg, we compared *cis*-eCpG, *distal*-eCpG and *trans*-eCpG mapping performance with and without a GPU. For CPU-based comparisons of the individual regions, computations were limited to a single core [11]. We also evaluated tecpg performance using a range of CPU core counts (i.e., 1, 2, 4, 8, 16, 24). These analyses used a dataset of whole blood samples collected from 333 participants (76% female) aged 18–78 years in the GTP (GSE72680, GSE58137). To facilitate the evaluation of the scaling performance of the GPU implementation when mapping *trans*-eCpGs across a wide range of eCpG counts, we sampled with replacement from the GTP dataset to obtain a sample size of 1000. All tecpg benchmarks were conducted on a physical server running Linux having 28 Xeon cores (2.3 GHz), 256 GB CPU memory, and a A2 GPU with 16 GB of memory (Nvidia Corporation, Santa Clara, CA).

## Results and discussion

**Fig. 1:**
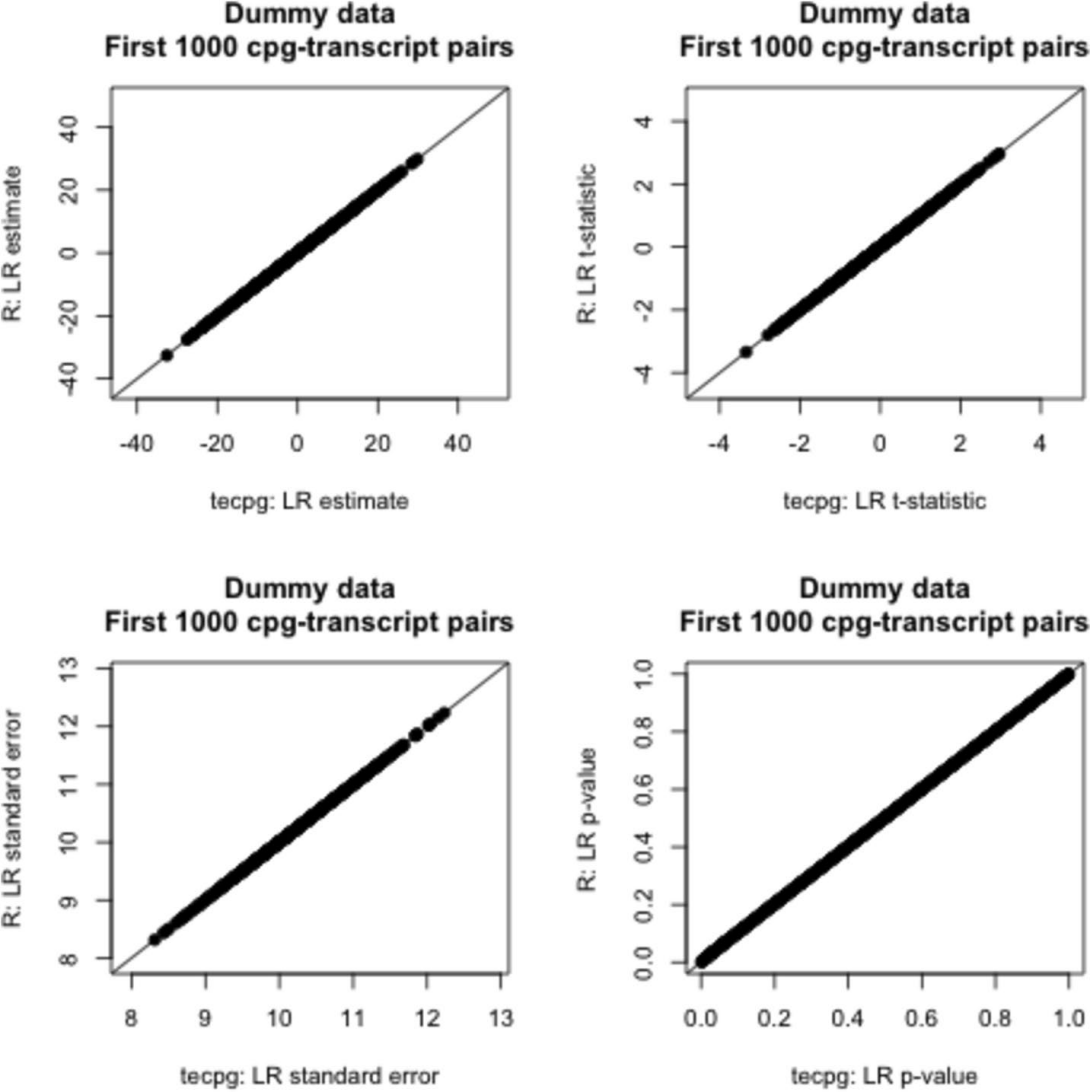
Comparisons of the first 1000 CpG-transcript pair linear regression analyses results between tecpg and lm() function in the stats package in R for a simulated dataset generated by sampling with replacement (n = 1000 samples). Simulated patient data was generated from real patient data in the Grady Trauma Project

To provide an open-source tool for eQTM mapping, we developed the Torch-eCpG software package. To evaluate the reproducibility of the linear regression analysis, we compared our results with those implemented in the lm() function in the stats package in R. As shown in Fig. 1, our implementation of the linear regression demonstrates high reproducibility.

**Fig. 2:**
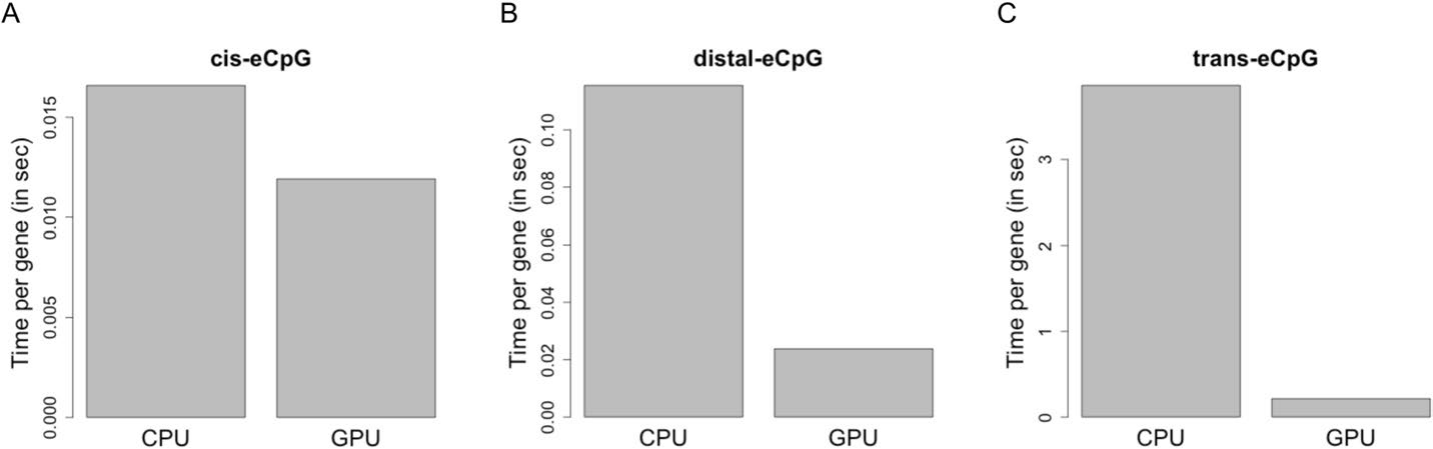
Performance of GPU implementations for eQTM mapping. Comparison of runtimes for tecpg analyses on CPU and GPU for **A** *cis*-eCpG, **B** *distal*-eCpG, and **C** *trans*-eCpG. The analyses evaluated 340 patients from the Grady Trauma Project dataset and included 422,442 methylation loci and 17,653 genes

**Fig. 3:**
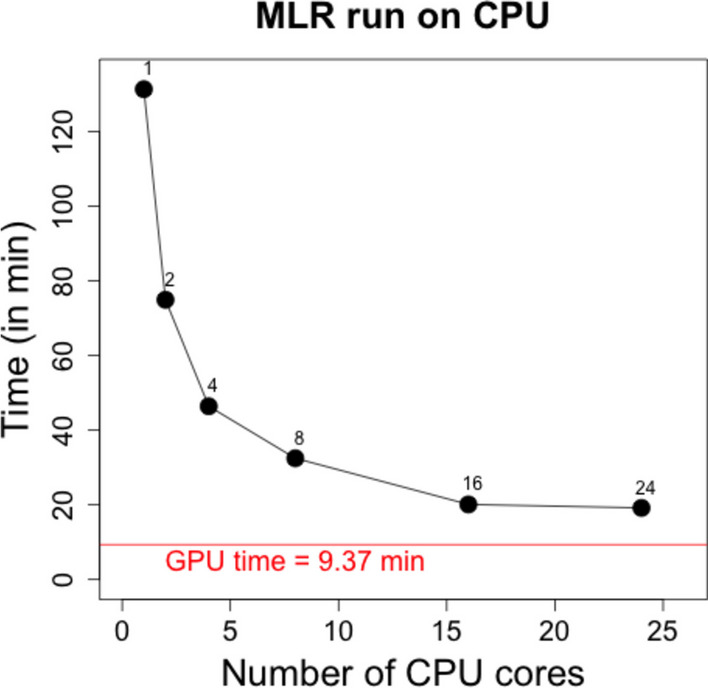
CPU runtimes for tecpg using 1, 2, 4, 8, 16, and 24 CPU cores. The analyses evaluated 340 patients from the Grady Trauma Project dataset and included 422,442 methylation loci and 17,653 genes

For tecpg benchmarking, we evaluated eQTM mapping using the CPU and GPU implementation in tecgp for 300 patients from the GTP patient dataset (422,442 methylation loci and 17,653 genes). Across the mapping modalities, the GPU outperformed the CPU analysis by up to 18x. Our implementation of the *cis-*eCpG mapping was 1.4 × faster on the GPU than that of the CPU (Fig. 2A). For *distal-*eCpG mapping, our implementation was 5 × faster on the GPU (Fig. 2B). Finally, for *trans*-eCpG mapping, our implementation was 18 × faster on the GPU (Fig. 2C). In terms of tecpg using additional cores, we found that major incremental improvements were realized by increasing the CPU core count up to 8, after which the gains were minimal (Fig. 3). Although the CPU performance did improve with additional cores, the GPU implementation was still 2 × faster than the 24-core CPU implementation.

**Fig. 4:**
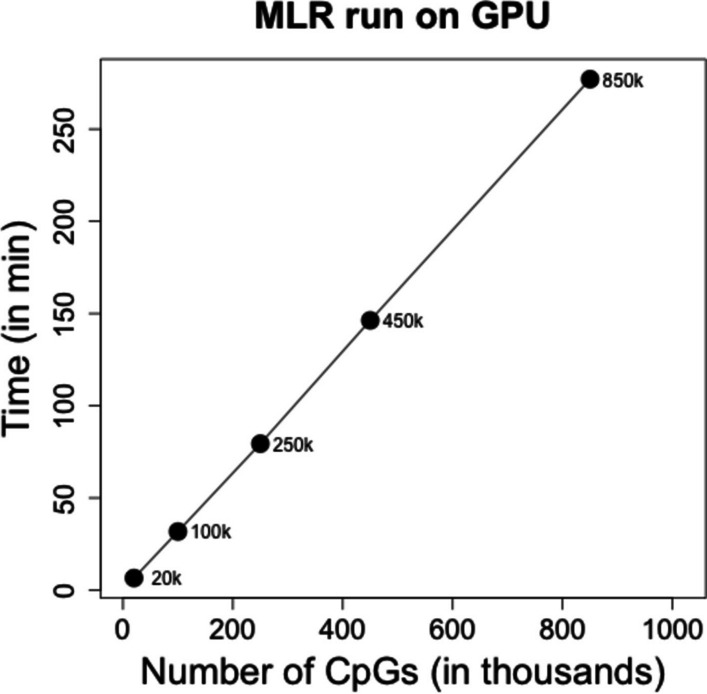
GPU runtime of tecpg for 1000 simulated patient samples for 20,000 genes and 20 × 10^3^, 100 × 10^3^, 250 × 10^3^, 450 × 10^3^ and 850 × 10^3^ CpG loci. Simulated patient data was generated from real patient data in the Grady Trauma Project. Data chunking was performed using 100 genes and 100,000 CpGs using 6.2 GB of GPU memory

**Fig. 5:**
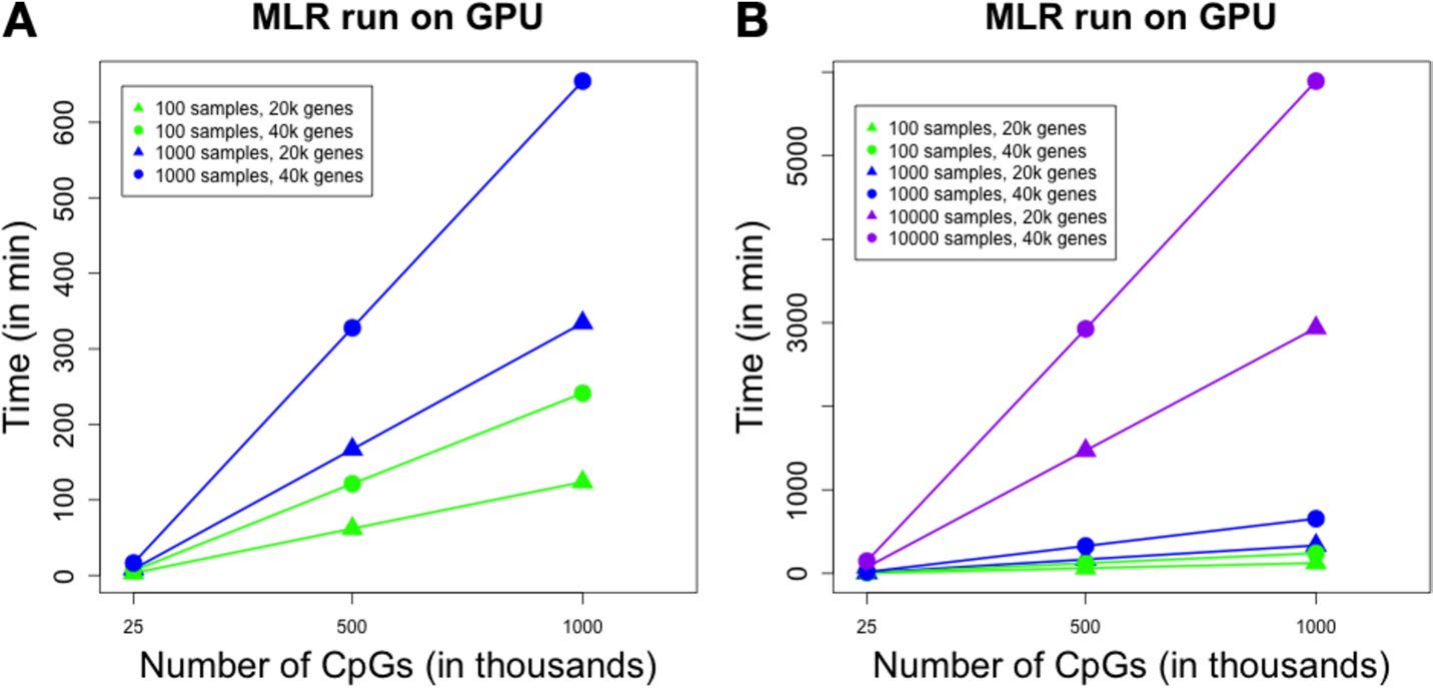
Runtime performance of Torch eCpG GPU implementation for the indicated number of samples, CpG loci, and gene expression loci. **A** n = 100 and n = 1000 samples. **B** n = 100, n = 1000, n = 10,000 samples. Simulated patient data was generated from real patient data in the Grady Trauma Project

We found tecpg scales linearly across a wide range of methylation loci for a reasonably large sample size (n = 1000 patients) (Fig. 4). In addition, the total time to evaluate 1000 patients for whole transcriptome (2 × 10^4^ genes) and whole methylome array data (8.5 × 10^5^) was < 15 h. The short time needed to evaluate a dataset sized to the largest currently available methylation array (i.e., Infinium MethylationEPIC) highlights the utility of this tool to evaluate eQTM mapping of dataset of realistic size. The linear scaling demonstrates the memory efficiency of the chunking of genes and CpG loci for analysis and is concordant with the embarrassingly parallel nature of this analysis (i.e., all gene × CpG loci comparisons are independent). This efficiency and scaling suggest the tool will be useful for larger datasets in the future (e.g., > 10,000 patients) (Table 1, Fig. 5) and is accessible to perform on smaller hardware setups (i.e., GPUs with smaller memory specifications).

**Table 1:** Runtime performance of Torch eCpG GPU implementation for the indicated number of samples, CpG loci, and gene expression loci. Data are plotted in Fig. 5

Number of samples	Number of CpG loci	Number of genes	Runtime (minutes)
100	25,000	40,000	6.29
100	500,000	40,000	121.42
100	1,000,000	40,000	241.24
100	25,000	20,000	3.06
100	500,000	20,000	62.04
100	1,000,000	20,000	123.80
1000	25,000	40,000	16.48
1000	500,000	40,000	327.85
1000	1,000,000	40,000	654.57
1000	25,000	20,000	8.35
1000	500,000	20,000	167.03
1000	1,000,000	20,000	334.29
10,000	25,000	40,000	147.18
10,000	500,000	40,000	2926.99
10,000	1,000,000	40,000	5892.98
10,000	25,000	20,000	73.57
10,000	500,000	20,000	1470.24
10,000	1,000,000	20,000	2937.30

Given the increased availability of whole genome bisulfite sequencing (WGBS) data, we fit a linear model to the range of methylation loci shown in Fig. 4 to estimate the time it would take to map larger datasets. With this model, we estimate it would take 6.35 days (9146.04 min) to complete an analysis of 28 million CpG loci for 20,000 genes from 10,000 patients. To evaluate this estimate empirically, we simulated a dataset of 28 million CpG loci for 20,000 genes from 10,000 patients. The major resource limitation to the analysis was the size of the input data, which is limited by the available CPU memory. The maximum resident set size of dataset with 100,000 and 1 million CpGs was approximately 4.8 GB and 20 GB of RAM, respectively. Larger CpG datasets (e.g., all 28 million CpGs) required memory resources outside the assumptions of a reasonably sized workstation (i.e., > 32 GB RAM). To manage the CPU memory usage to store the datasets in memory prior to analysis, the total dataset was split into smaller pieces, with each split of data using approximately 20 GB of RAM to load. To manage the GPU memory during analysis, we set the chunking sizes of 100 genes and 100,000 CpGs. The GPU memory usage was 6.2 GB. With this approach, the analysis of a simulated WGBS dataset completed in 6.75 days, similar to our estimated time for completion.

## Conclusions

Torch-eCpG is the first freely available open-source tool to perform eQTM mapping. It provides a scalable and high-performance implementation that supports GPU enabled systems. By reducing computing time the tool offers cost-savings on shared systems (e.g., clusters) or cloud-based computing resources that charge by units of time. This tool allows for individual research labs with limited computational resources to perform analyses on affordable computer equipment or cloud-based virtual machines.

### Availability and requirements

- Project name: Torch-eCpG
- Project home page: http://www.github.com/kordk/torch-ecpg
- Operating system(s): Platform independent
- Programming language: Python 3.10 or higher
- Other requirements: click ~= 8.0.3, colorama ~= 0.4.4, matplotlib ~= 3.5.1, numpy ~= 1.24.1, pandas ~= 1.3.5, psutil ~= 5.9.4, requests ~  = 2.26.0, scipy ~= 1.10.0, setuptools ~= 63.3.0, torch ~= 1.13.1 + cu116
- License: BSD-3-Clause
- Any restrictions to use by non-academics: license needed.

## Data Availability

The datasets analyzed in this study are publicly available in the Gene Expression Omnibus repository (https://www.ncbi.nlm.nih.gov/geo/query/acc.cgi?acc=GSE72680 and https://www.ncbi.nlm.nih.gov/geo/query/acc.cgi?acc=GSE58137).

## Abbreviations

BED: Browser extensible data
CDF: Cumulative distribution function
CpG: Cytosine phosphate guanine
CPU: Central processing unit
CSV: Comma separated value
DNA: Deoxyribonucleic acid
eCpG: Expression associated CpG
eQTM: Expression quantitative trait methylation
GEO: Gene expression omnibus
GB: Gigabyte
GHz: Gigahertz
GPU: Graphical processing unit
GTP: Grady trauma project
